# Association between early cognitive rehabilitation and long-term cognitive recovery in patients with mild traumatic brain injury: a retrospective cohort study

**DOI:** 10.3389/fnhum.2026.1846518

**Published:** 2026-07-13

**Authors:** Yi Ren, Huiying Wu, Yumei Lu, Qi Zeng, Qian Su

**Affiliations:** 1 School of Life Sciences and Technology, Xi’an Jiaotong University, Xi’an, Shaanxi, China; 2 Department of Rehabilitation Medicine, Hanzhong Hospital of TCM, Hanzhong, Shaanxi, China

**Keywords:** cognitive recovery, early cognitive rehabilitation (E-CR), functional outcomes, inflammation, mild traumatic brain injury (mTBI), neuroinjury biomarkers

## Abstract

**Objective:**

To examine the association between early cognitive rehabilitation (E-CR) and long-term cognitive, functional, and biomarker outcomes in patients with mild traumatic brain injury (mTBI).

**Methods:**

This single-center retrospective cohort study included 185 patients with mTBI who completed at least 12 months of follow-up. E-CR was defined as at least three cognitive rehabilitation sessions within 30 days after injury, whereas patients without rehabilitation within 30 days or with rehabilitation initiated after 30 days were classified as the Non-early-CR group. Cognitive outcomes were assessed using MoCA scores at 6 and 12 months, and functional outcomes included ADL, GOS-E, and return to work or study. Serum NfL, GFAP, and IL-6 were longitudinally analyzed as exploratory biomarkers.

**Results:**

Among 185 patients, 91 were included in the E-CR group and 94 in the Non-early-CR group. Baseline characteristics were comparable between groups. At 12 months, the E-CR group showed greater MoCA improvement than the Non-early-CR group (ΔMoCA: 7.03 vs. 3.01, *p* < 0.001) and a higher proportion of patients achieving MoCA ≥26 (34.1% vs. 7.4%). E-CR remained independently associated with MoCA improvement after adjustment for age, education, admission GCS score, and baseline MoCA score. The E-CR group also showed better ADL and GOS-E outcomes. Exploratory biomarker analyses showed lower NfL, GFAP, and IL-6 levels in the E-CR group at 6 and 12 months.

**Conclusion:**

E-CR was associated with better long-term cognitive and functional outcomes and with more favorable exploratory biomarker trajectories in patients with mTBI. Prospective studies are needed to confirm causality and clarify underlying mechanisms.

## 1 Introduction

The majority of patients with mild traumatic brain injury (mTBI) recover fully within days to weeks (Wu et al., 1990; Haarbauer-Krupa et al., 2021; Godbolt et al., 2014). However, approximately 15–30% experience persistent cognitive disturbances lasting months or longer, despite the absence of overt structural abnormalities on routine neuroimaging (Jourdan et al., 2018; Humphries et al., 2020). The neurobiological substrates of these prolonged impairments involve diffuse axonal injury, synaptic dysfunction, and sustained neuroinflammation (Aungst et al., 2014; Graham et al., 2020; Lozano et al., 2015), challenging the traditional perception of mTBI as a uniformly benign and self-limiting condition and highlighting the need for effective early interventions.

From a neuroplasticity standpoint, the early post-injury period may represent a critical therapeutic window during which external stimulation can promote synaptic remodeling and mitigate secondary neuroinflammatory cascades (Ramanathan et al., 2019; Zotey et al., 2023; Mohamadpour et al., 2019). Preclinical and neuroimaging studies demonstrate that targeted cognitive engagement can facilitate functional reorganization of key networks disrupted after mTBI (Wu et al., 2020; Gimbel et al., 2021), providing a biological rationale for early cognitive rehabilitation (CR).

Clinically, however, the management of cognitive symptoms after mTBI remains mostly observational or symptomatic. Although structured cognitive rehabilitation has shown benefit in moderate-to-severe TBI populations (Galetto and Sacco, 2017), its role in mTBI is far less defined. Patients rarely receive early intervention due to mild initial presentation, insufficient follow-up systems, or lack of standardized rehabilitation pathways, causing many to miss the critical plasticity window. As a result, 15–30% of individuals continue to experience long-term cognitive, emotional, and social limitations 6–12 months after injury (Wu et al., 1990; Haarbauer-Krupa et al., 2021; Godbolt et al., 2014; Jourdan et al., 2018; Pease et al., 2024). These persistent deficits, which are often undetectable on routine clinical examination yet profoundly disabling, highlight an important gap between mechanistic understanding and real-world clinical practice.

Despite the strong biological rationale and compelling clinical need, high-quality evidence supporting the effectiveness of early structured cognitive rehabilitation in mild traumatic brain injury remains scarce, especially in real-world clinical settings. Moreover, the neurobiological correlates of rehabilitation-related recovery have been insufficiently investigated. Whether early CR is associated with faster resolution of neural injury markers such as NfL, GFAP, and IL-6, and whether these biomarker trajectories parallel changes in cognitive performance, remains largely unknown.

To bridge this gap, we conducted a retrospective cohort study to examine the association between early cognitive rehabilitation, initiated within 30 days of injury, and cognitive recovery, daily functioning, and social reintegration in patients with mTBI. As a secondary exploratory aim, we longitudinally assessed serum NfL, GFAP, and IL-6 levels to determine whether biomarker trajectories paralleled cognitive improvement.

## 2 Methods

### 2.1 Study design

This single-center retrospective cohort study included patients hospitalized at Hanzhong Traditional Chinese Medicine Hospital with a diagnosis of mild traumatic brain injury, defined as a Glasgow Coma Scale score of 13–15, between July 2023 and June 2025. All included patients completed at least 12 months of follow-up. The study was approved by the Ethics Committee of Hanzhong Traditional Chinese Medicine Hospital (approval number: HZZYY2023002) and was conducted in accordance with the principles of the Declaration of Helsinki.

### 2.2 Participants

Eligible patients met all of the following conditions: (1) Age ≥18 years; (2) Clinically diagnosed with mild traumatic brain injury (mTBI) at admission, with a Glasgow Coma Scale (GCS) score of 13–15 and meeting the American Congress of Rehabilitation Medicine (ACRM) criteria for mTBI, including at least one of the following: loss of consciousness ≤30 min, post-traumatic amnesia ≤24 h, or alteration of mental state at the time of injury (Pease et al., 2024); (3) No skull fractures, intracranial hemorrhage, or history of neurosurgical intervention at admission; (4) No prior history of central nervous system disorders, psychiatric illness, or intellectual disability; (5) Complete inpatient medical records and at least 6 months of follow-up; (6) Underwent at least one cognitive assessment using the Montreal Cognitive Assessment (MoCA) during follow-up.

Patients were excluded if they met any of the following: (1) Died during hospitalization, discharged against medical advice, or lost to follow-up; (2) Inability to determine whether cognitive rehabilitation was administered; (3) Incomplete follow-up data or missing MoCA scores; (4) Presence of severe polytrauma or concurrent neurosurgical treatment.

All patients underwent non-contrast head CT within 24 h of injury, and scans were reviewed by board-certified radiologists as part of routine clinical care. Patients with skull fracture, intracranial hemorrhage, cerebral contusion, edema, or neurosurgical indications were excluded. Brain MRI was not routinely performed in this retrospective cohort. Available medical records showed no documented history of prior significant head trauma, recurrent mTBI, or repeated head injury among included patients. Only minor non-hemorrhagic extracranial findings, such as scalp swelling or soft tissue changes, were recorded as minor CT findings.

### 2.3 Intervention and grouping

Patients were divided into an early cognitive rehabilitation group (E-CR group) and a control group (Non-early-CR Group) based on whether they received cognitive rehabilitation within 30 days post-injury. The E-CR group included patients who completed at least three sessions of cognitive rehabilitation during this early period, while the Non-early-CR group included those who either received no cognitive rehabilitation within 30 days post-injury or initiated it after 30 days post-injury.

Cognitive rehabilitation was provided by licensed rehabilitation therapists using individualized protocols targeting core cognitive domains such as memory, language, attention, and executive function. Intervention modalities included occupational therapy, language or attention training, and computer-assisted cognitive programs. Module selection was guided by the patient’s baseline MoCA sub-domain profile, self-reported cognitive complaints, and the treating therapist’s clinical judgment, with the goal of targeting the most impaired or functionally relevant cognitive domains for each individual. Each patient typically participated in 2 to 4 core modules. Sessions lasted 20 to 45 min and were delivered 2 to 5 times per week. The total duration and frequency of intervention were adjusted based on the patient’s clinical condition and response to maximize therapeutic benefit (Cicerone et al., 2019; Kennedy et al., 2022).

### 2.4 Variables and outcome measures

The baseline characteristics of the patients included age (years), sex (male/female), years of education, Glasgow Coma Scale (GCS) score at admission, presence of minor non-hemorrhagic findings on head CT, length of hospital stay (days), use of sedative or antidepressant medications (yes/no), and presence of comorbid conditions such as hypertension or diabetes. For patients in the intervention group, the time from injury to the initiation of cognitive rehabilitation was also recorded. Intervention variables included the total number of cognitive rehabilitation sessions (categorized as ≤4, 5–7, or ≥8 sessions) and the type of training received, including memory training, language training, attention training, and executive function training.

The primary outcome was the improvement in the total score of the MoCA at 6 and 12 months post-discharge. The MoCA assesses eight cognitive domains, with a maximum score of 30. A score ≥26 is considered normal; scores between 18 and 25 indicate mild cognitive impairment, 10–17 indicate moderate impairment, and <10 indicate severe impairment (Hobson, 2015; Liu et al., 2023). The change in MoCA score (ΔMoCA) was calculated by subtracting the baseline score from the follow-up score. To ensure assessment objectivity, follow-up MoCA tests were administered by a trained neuropsychologist who was blinded to group allocation and not involved in the delivery of rehabilitation services.

Secondary outcomes included activities of daily living (ADL), global functional status, and return to work or study. ADL was assessed using the Barthel Index, which includes 10 items covering essential self-care tasks. Each item is scored using one of three scales: (0, 5), (0, 5, 10), or (0, 5, 10, 15), with a maximum total score of 100. Higher scores indicate greater independence, and scores ≥90 are considered indicative of functional independence (Doussoulin et al., 2020). Global functional outcomes were assessed using the Glasgow Outcome Scale-Extended (GOS-E), with a score ≥5 indicating a favorable outcome (Yu et al., 2025).

### 2.5 Serum biomarker assessment

To investigate the potential neurobiological correlates associated with early cognitive rehabilitation in patients with mild traumatic brain injury (mTBI), serum samples were collected from all enrolled patients for the measurement of three brain injury-related biomarkers: neurofilament light chain (NfL), glial fibrillary acidic protein (GFAP), and interleukin-6 (IL-6). No separate biomarker subgroup was selected; biomarker analyses were performed in the full cohort of 185 patients with valid longitudinal serum measurements at the predefined time points.

Blood samples were collected at three predefined time points: T1 (baseline, within 30 days post-injury and prior to any cognitive intervention), T2 (at 6-month follow-up), and T3 (at 12-month follow-up). Following centrifugation at 3,000 g for 10 min, serum was aliquoted into 0.5 mL cryotubes and stored at −80 °C. Only samples with ≤1 freeze–thaw cycle and no visible hemolysis were included in the final analysis. All biomarkers were measured using the Simoa platform or other immunoassay techniques with comparable sensitivity. Each batch included high-, medium-, and low-level quality control samples, as well as blanks. Intra-assay and inter-assay coefficients of variation (CVs) were maintained below 10 and 15%, respectively. The time from blood draw to freezing and the number of freeze–thaw cycles were recorded for all specimens.

### 2.6 Follow-up

Follow-up assessments were conducted as part of routine clinical care through scheduled rehabilitation clinic visits. Referral to cognitive rehabilitation was triggered by the attending physician based on clinical judgment, typically in response to patient-reported cognitive complaints or observed cognitive difficulties during the acute inpatient stay. All follow-up assessments, including MoCA testing and functional evaluations, were performed during these routine clinical visits rather than through a separate research protocol.

### 2.7 Statistical analysis

All analyses were performed using SPSS 23.0 Continuous variables were compared using independent or paired Student’s t-tests, and categorical variables using chi-square or Fisher’s exact tests. MoCA score changes (ΔMoCA) were analyzed within and between groups. Multivariable linear regression was used to assess the association between early cognitive rehabilitation and MoCA improvement, adjusting for age, years of education, admission GCS score, and baseline MoCA score. Because of the retrospective design, these models were intended to reduce measured confounding. Dose–response trends were tested using trend tests (P for trend) to evaluate whether a greater number of cognitive rehabilitation sessions was associated with a larger improvement in MoCA scores. Pearson correlations were also calculated to examine the relationship between the number of sessions and MoCA improvement. A two-sided *p* value <0.05 was considered statistically significant.

Biomarker data were analyzed using linear mixed-effects models (LMM) to assess the effects of group and time on NfL, GFAP, and IL-6 levels. A Group × Time interaction was tested to determine whether the rate of change in biomarkers differed between the E-CR and Non-early-CR groups. *Post-hoc* pairwise comparisons were made to assess differences between time points, and the Holm-Bonferroni method was applied to correct for multiple comparisons.

## 3 Results

### 3.1 Baseline characteristics of patients

A total of 185 patients with mild traumatic brain injury (mTBI) who met the inclusion criteria were included in the final analysis, comprising 91 cases in the E-CR group and 94 cases in the Non-early-CR group. All patients completed at least 12 months of follow-up with complete clinical data and MoCA assessments.

To evaluate comparability between groups, baseline demographic and clinical characteristics were analyzed. No significant differences were found between the two groups (*p* > 0.05) in the baseline variables listed in Table 1, including age, sex distribution, years of education, baseline MoCA score, admission GCS score, and presence of minor non-hemorrhagic CT findings.

**Table 1:** Baseline characteristics of patients in the E-CR and Non-early-CR groups.

Variable	E-CR group	Non-early-CR group	Test statistic	*p* value
	(*n* = 91)	(*n* = 94)		
Age (years), mean ± SD	50.21 ± 19.49	47.63 ± 17.86	t = 0.94	0.349
Sex, *n* (%)			χ^2^ = 0.01	0.942
Male	46 (50.5)	46 (48.9)		
Female	45 (49.5)	48 (51.1)		
Years of education, mean ± SD	8.80 ± 6.51	9.39 ± 6.08	t = −0.64	0.524
Baseline MoCA score, mean ± SD	14.97 ± 5.80	13.90 ± 5.30	t = 1.31	0.195
GCS at admission, mean ± SD	13.99 ± 0.78	14.05 ± 0.82	t = −0.54	0.587
Minor CT findings, *n* (%)			χ^2^ = 0.90	0.344
Yes	44 (48.4)	53 (56.4)		

The average time to initiation of cognitive rehabilitation in the E-CR group was 4.85 ± 2.54 days post-injury, with all patients receiving treatment within 30 days, consistent with the study’s definition of early intervention.

Overall, the two groups were well matched in terms of baseline demographic and clinical characteristics, ensuring comparability for subsequent outcome analyses.

### 3.2 Changes in MoCA scores and proportion of normal cognition

Based on the confirmed baseline comparability between groups, we further analyzed changes in cognitive function during the follow-up period.

**Figure 1:**
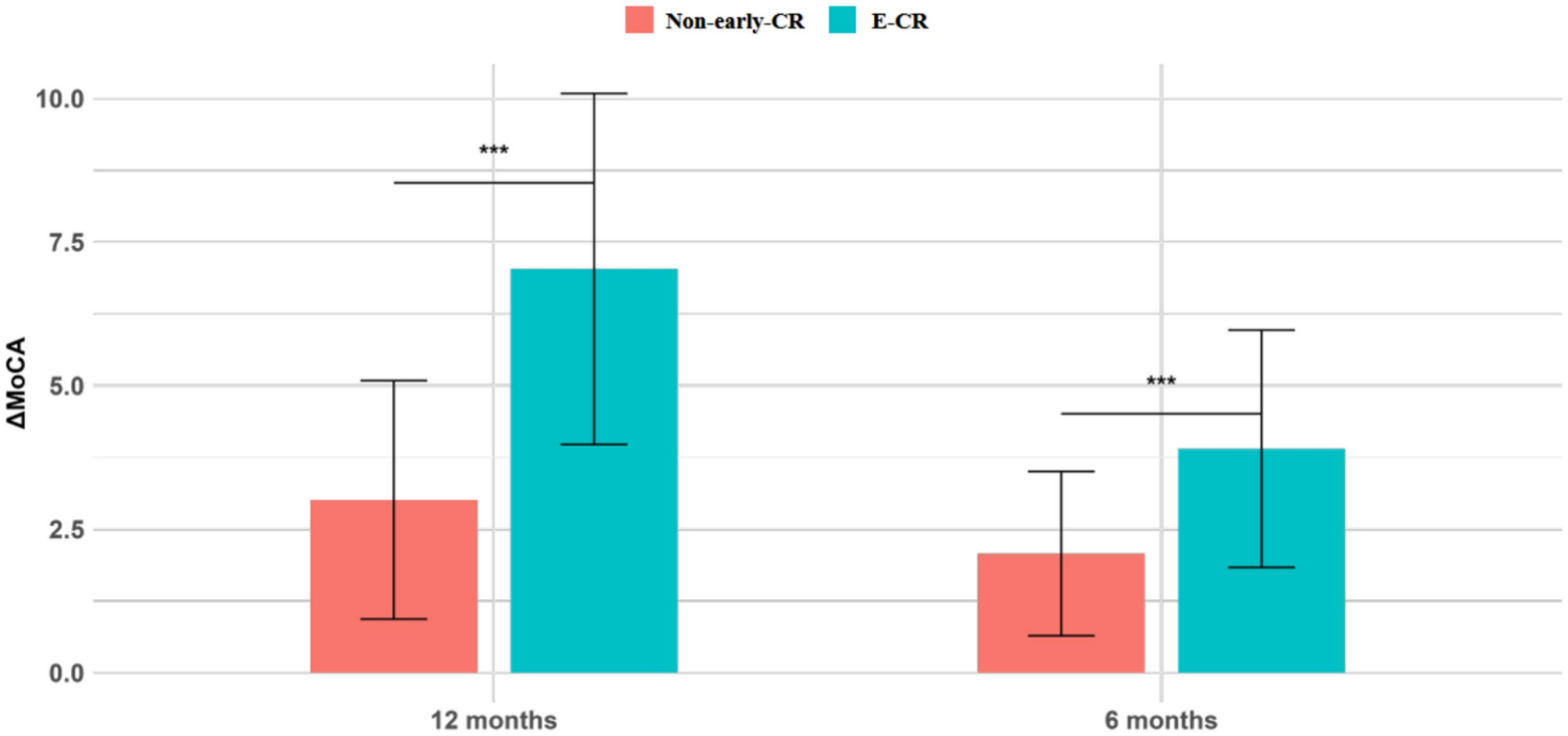
Comparison of MoCA score improvement between the E-CR and Non-early-CR groups at 12-month follow-up. ***p < 0.001 vs. the Non-early-CR group.

As shown in Table 2, the E-CR group consistently demonstrated significantly higher MoCA scores compared to the Non-early-CR group at both follow-up time points. At 6 months post-discharge, the MoCA score was 18.87 ± 5.80 in the E-CR group and 15.98 ± 5.56 in the Non-early-CR group (*p* < 0.001). By 12 months, the gap widened further to 22.00 ± 5.99 vs. 16.91 ± 5.57, respectively (*p* < 0.001). Additionally, the degree of MoCA improvement from baseline was more substantial in the E-CR group (Figure 1), with a mean change of 7.03 ± 3.06 points at 12 months, compared to only 3.01 ± 2.08 points in the Non-early-CR group (*p* < 0.001).

**Table 2:** MoCA scores and proportion of cognitive normalization in the E-CR and Non-early-CR groups.

Variable	E-CR group (*n* = 91)	Non-early-CR group (*n* = 94)	*p* value
MoCA score at 6 months, Mean ± SD	18.87 ± 5.80	15.98 ± 5.56	0.00068
MoCA score at 12 months, Mean ± SD	22.00 ± 5.99	16.91 ± 5.57	<0.00001
MoCA ≥26 at 6 months, *n* (%)	13.20%	4.30%	0.05755
MoCA ≥26 at 12 months, *n* (%)	34.10%	7.40%	0.00002

We also assessed the proportion of patients who achieved a MoCA score ≥26, indicating recovery to a normal cognitive level. At 6 months, the rate was 13.2% in the E-CR group vs. 4.3% in the Non-early-CR group. At 12 months, the E-CR group showed a markedly higher recovery rate of 34.1% compared to 7.4% in the Non-early-CR group.

These findings suggest that early cognitive rehabilitation was associated with sustained improvement in cognitive function and a higher likelihood of cognitive normalization in patients with mTBI.

### 3.3 Multivariable analysis of MoCA score improvement

To determine the independent association between early cognitive rehabilitation and MoCA score improvement, a multivariable linear regression model was constructed. The change in MoCA score at 12 months (ΔMoCA) was used as the dependent variable, with group (E-CR vs. Non-early-CR Group), age, years of education, admission GCS score, and baseline MoCA score included as covariates.

Results showed that early cognitive rehabilitation was significantly associated with greater MoCA improvement. After adjusting for all covariates, patients in the E-CR group had an average increase of 4.08 points in ΔMoCA compared with the Non-early-CR group (*β* = 4.079, 95% CI: 3.321 to 4.836, *p* < 0.001). In contrast, age (*p* = 0.672), years of education (*p* = 0.293), GCS score (*p* = 0.218), and baseline MoCA score (*p* = 0.067) were not statistically significant predictors (Table 3).

**Table 3:** Multivariable linear regression analysis of MoCA score improvement at 12 months.

Variable	Coefficient (β)	95% CI	*p* value
Early cognitive rehabilitation (Yes vs. No)	4.079	3.321 to 4.836	<0.001
Years of education (per 1 year increase)	−0.032	−0.093 to 0.028	0.293
Age (per 1 year increase)	0.004	−0.016 to 0.025	0.672
Admission GCS score	0.296	−0.176 to 0.767	0.218
Baseline MoCA score	−0.064	−0.132 to 0.004	0.067

In a further subgroup analysis within the E-CR group, the number of days from injury to initiation of cognitive rehabilitation was included as a continuous variable in the model. This variable was significantly negatively associated with MoCA improvement (*β* = −0.494, 95% CI: −0.737 to −0.251, *p* < 0.001), indicating that earlier initiation of rehabilitation was associated with better cognitive outcomes.

### 3.4 Functional outcomes and social reintegration

At the 12-month follow-up, patients in the E-CR group demonstrated better outcomes in activities of daily living, global functional status, and social reintegration compared to those in the Non-early-CR group (Table 4).

**Table 4:** Comparison of functional and social outcomes between the E-CR and Non-early-CR groups at 12 months.

Variable	E-CR group (*n* = 91)	Non-early-CR group (*n* = 94)	Test statistic	*p* value
ADL score at 12 months, Mean ± SD	90.56 ± 7.74	74.28 ± 9.80	t = 12.564	< 2.2 × 10^−16^
ADL ≥ 90, *n* (%)	54.9	6.4	χ^2^ = 49.385	2.1 × 10^−12^
GOS-E ≥ 5, *n* (%)	61.5	28.7	χ^2^ = 18.824	1.4 × 10^−5^
Return to work or study, *n* (%)	53.8	40.4	χ^2^ = 2.826	0.0928

The average ADL score was significantly higher in the E-CR group (90.56 ± 7.74) than in the Non-early-CR group (74.28 ± 9.80, *p* < 0.001). Additionally, a significantly greater proportion of patients in the E-CR group achieved an ADL score ≥90, indicating independent living ability (54.9% vs. 6.4%, *p* < 0.001).

For global functional outcome, 61.5% of patients in the E-CR group reached a favorable outcome (GOS-E ≥ 5), compared to 28.7% in the Non-early-CR group (*p* < 0.001).

Regarding return to work or study, the E-CR group showed a higher recovery rate (53.8% vs. 40.4%), though the difference was not statistically significant (*p* = 0.0928).

These findings suggest that early cognitive rehabilitation was associated not only with better cognitive function but also with greater daily functional independence and more favorable global functional outcomes in patients with mTBI, with a possible association with social reintegration.

### 3.5 Dose–response relationship between rehabilitation frequency and MoCA improvement

To explore the dose–response relationship between cognitive rehabilitation frequency and cognitive improvement, patients in the E-CR group were categorized into three subgroups based on the total number of cognitive training sessions: ≤4 sessions (*n* = 33), 5–7 sessions (*n* = 31), and ≥8 sessions (*n* = 27).

As shown in the analysis, MoCA score improvement increased significantly with higher intervention frequency (P for trend = 1.81 × 10^−7^). At 12 months, the ≥8 sessions group demonstrated the greatest improvement (mean ΔMoCA: 9.30 ± 3.53), significantly higher than the 5–7 sessions group (6.81 ± 2.29) and the ≤4 sessions group (5.39 ± 2.03, *p* = 8.69 × 10^−7^) (Table 5).

**Figure 2:**
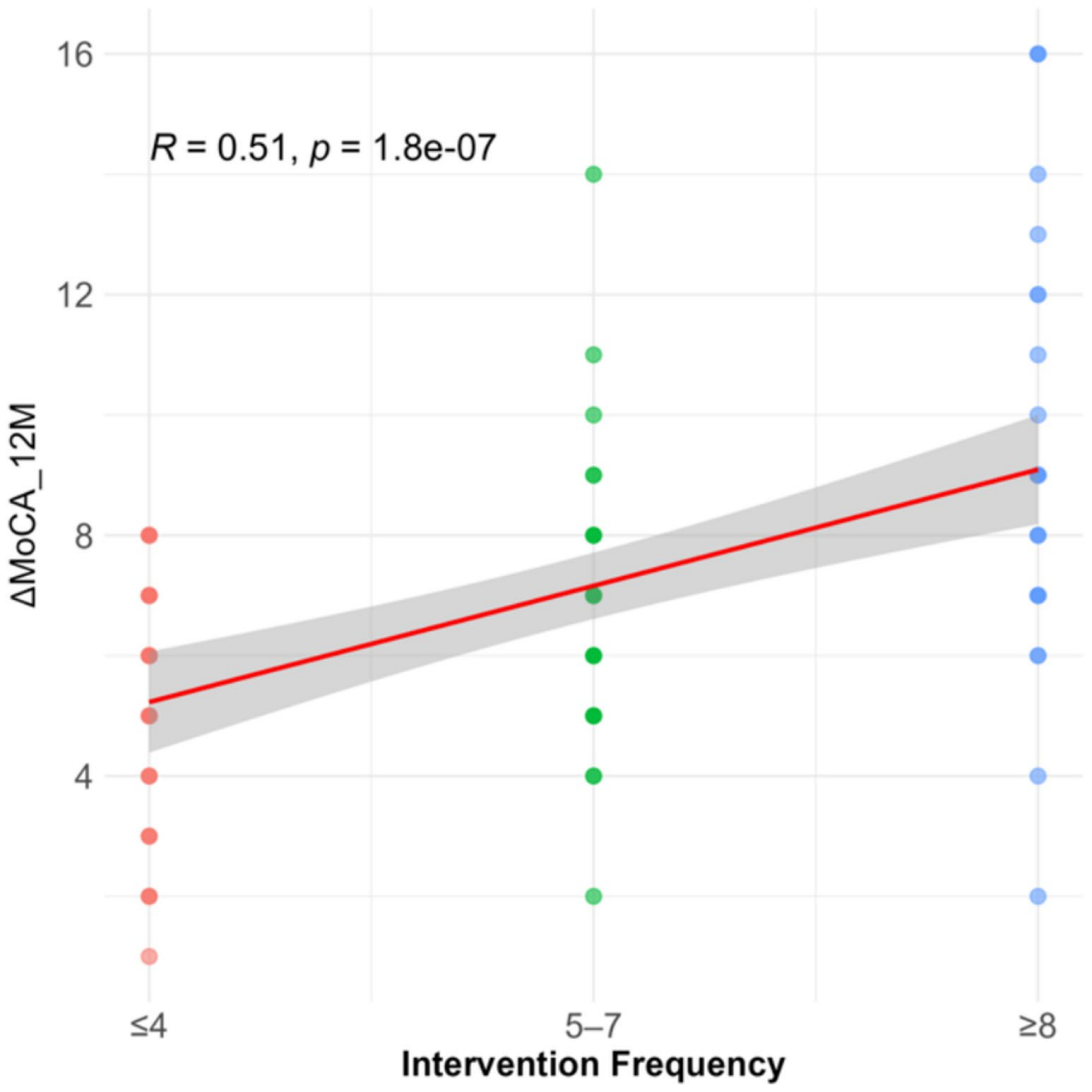
Correlation between the number of cognitive rehabilitation sessions and MoCA score improvement.

Pearson correlation analysis showed a moderate positive correlation between the number of sessions and MoCA improvement (r = 0.51, *p* = 1.81 × 10^−7^) (Figure 2).

These results suggest a clear dose–response relationship, indicating that more frequent cognitive rehabilitation is associated with greater cognitive gains in patients with mTBI.

**Table 5:** Relationship between number of cognitive rehabilitation sessions and MoCA improvement.

Session group	Sample size (*n*)	MoCA improvement (Δ score, Mean ± SD)
≤4 sessions	33	5.39 ± 2.03
5–7 sessions	31	6.81 ± 2.29
≥8 sessions	27	9.30 ± 3.53
P for trend	—	1.81 × 10^−7^

### 3.6 Association between different rehabilitation modalities and MoCA improvement

To evaluate the potential association between different cognitive training modalities and cognitive improvement, a subgroup analysis was conducted within the E-CR group based on the primary type of intervention received. Among the patients, 23.1% (*n* = 21) primarily received memory training, 24.2% (*n* = 22) language training, 33.0% (*n* = 30) attention training, and 19.8% (*n* = 18) executive function training. As shown in Table 6, there were no statistically significant differences in MoCA improvement across the four subgroups at either the 6-month or 12-month follow-up.

**Table 6:** Comparison of MoCA improvement across different cognitive training modalities.

Intervention type	*n*	ΔMoCA at 6 months (Mean ± SD)	*Δ*MoCA at 12 months (Mean ± SD)
Memory training	21	3.71 ± 1.93	7.29 ± 2.74
Language training	22	3.82 ± 1.87	6.91 ± 2.74
Attention training	30	4.23 ± 2.37	7.77 ± 3.49
Executive function training	18	3.67 ± 2.00	5.67 ± 2.74
Total	91	*F* = 0.397, *p* = 0.756	*F* = 1.889, *p* = 0.137

These findings suggest that different types of cognitive training were associated with cognitive improvement under a structured but individualized rehabilitation framework. However, no specific modality demonstrated clear superiority.

### 3.7 Between-group differences in serum biomarkers

**Figure 3:**
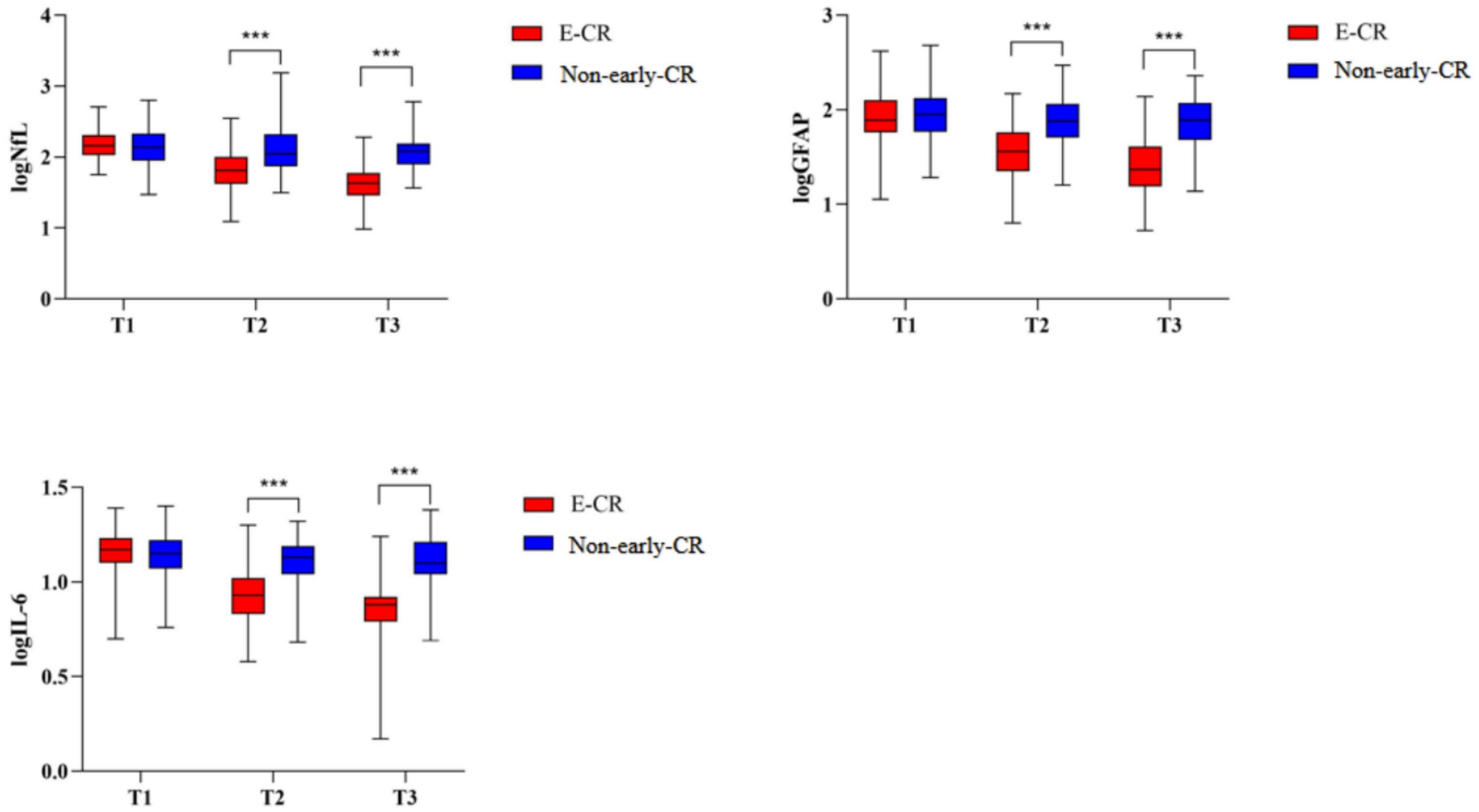
Longitudinal box plots of logNfL, logGFAP, and logIL-6 levels in E-CR and Non-early-CR groups.

As a secondary, exploratory aim, we next examined whether the two groups showed distinct biological profiles over time. Figure 3 illustrates the distribution of logNfL, logGFAP, and logIL-6 at each time point, with corresponding values listed in Table 7.

**Table 7:** Between-group comparison of serum biomarker levels at T1, T2, and T3.

Biomarker	Group	*n*	T1	T2	T3
logNfL	E-CR	91	2.15 ± 0.21	1.82 ± 0.30	1.63 ± 0.24
	Non-early-CR Group	94	2.14 ± 0.28	2.11 ± 0.30	2.07 ± 0.24
	t		0.242	−6.553	−12.534
	P		0.809	<0.001	<0.001
logGFAP	E-CR	91	1.90 ± 0.29	1.55 ± 0.28	1.38 ± 0.3
	Non-early-CR Group	94	1.94 ± 0.28	1.88 ± 0.27	1.87 ± 0.27
	t		−0.945	−7.954	−11.629
	P		0.346	<0.001	<0.001
logIL-6	E-CR	91	1.15 ± 0.12	0.94 ± 0.14	0.86 ± 0.13
	Non-early-CR Group	94	1.14 ± 0.11	1.11 ± 0.12	1.10 ± 0.13
	t		0.682	−8.985	−12.438
	P		0.496	<0.001	<0.001

NfL, GFAP, and IL-6 concentrations were originally measured in pg/mL and log-transformed before statistical analysis to improve distribution normality. Values shown represent log-transformed biomarker levels.

At baseline (T1), biomarker levels did not differ between groups (all *p* > 0.05), indicating comparable initial neuroaxonal injury and inflammatory status. By 6 months (T2), clear divergence emerged: the E-CR group showed significantly lower logNfL (1.82 ± 0.30 vs. 2.11 ± 0.30), logGFAP (1.55 ± 0.28 vs. 1.88 ± 0.27), and logIL-6 (0.94 ± 0.14 vs. 1.11 ± 0.12), all *p* < 0.001. These differences further widened at 12 months (T3), with the E-CR group maintaining consistently lower levels of logNfL (1.63 ± 0.24 vs. 2.07 ± 0.24), logGFAP (1.38 ± 0.30 vs. 1.87 ± 0.27), and logIL-6 (0.86 ± 0.13 vs. 1.10 ± 0.13), again *p* < 0.001.

These findings indicate that early cognitive rehabilitation was associated with a more pronounced reduction in neuroaxonal injury and inflammatory markers, consistent with the greater clinical improvement observed in the E-CR group.

### 3.8 Longitudinal changes in serum biomarkers

Building on the group differences described above, the longitudinal mixed-effects model further confirmed that biomarker trajectories differed significantly between groups. As shown in Table 8, the Group × Time interaction was significant for logNfL (*F* = 26.025, *p* < 0.001), logGFAP (*F* = 21.500, *p* < 0.001), and logIL-6 (*F* = 12.945, *p* < 0.001), indicating a faster decline in the E-CR group over time.

**Table 8:** Linear mixed-effects model analysis of longitudinal changes in biomarkers.

Biomarker	Group		Time		Group × Time		Rehabilitation sessions	
	F	P	F	P	F	P	F	P
logNfL	28.779	<0.001	39.070	<0.001	26.025	<0.001	5.705	0.017
logGFAP	28.839	<0.001	34.248	<0.001	21.500	<0.001	4.123	0.043
logIL-6	2.607	0.107	25.352	<0.001	12.945	<0.001	4.284	0.039

The main effect of Time was also significant across all three markers (all *p* < 0.001), and the Group effect remained significant for logNfL and logGFAP (both *F* > 28, *p* < 0.001). Rehabilitation frequency showed a modest but significant negative association with biomarker levels (all *p* < 0.05), suggesting greater reductions with more training sessions.

In summary, early cognitive rehabilitation was associated with steeper longitudinal changes in biomarker levels, suggesting a faster biomarker decline pattern in the E-CR group.

### 3.9 *Post-hoc* pairwise comparisons

To clarify the specific time-point differences underlying the significant Group × Time interactions reported in Section 3.8, *post-hoc* pairwise analyses were performed. The detailed results are summarized in Table 9.

**Table 9:** Post-hoc pairwise comparisons.

Comparison type	Time point comparison	logNfL	logGFAP	logIL-6
Within-group comparisons	E-CR			
	T1 vs. T2	0.419 (0.292–0.546), <0.001	0.42 (0.285–0.555), <0.001	0.174 (0.114–0.235), <0.001
	T1 vs. T3	0.677 (0.494–0.861), <0.001	0.656 (0.462–0.849), <0.001	0.228 (0.141–0.316), <0.001
	T2 vs. T3	0.259 (0.141–0.377), <0.001	0.236 (0.111–0.36), <0.001	0.054 (−0.002–0.11), 0.062
	Non-early-CR Group			
	T1 vs. T2	0.036 (−0.056–0.128), 1	0.06 (−0.039–0.159), 0.428	0.026 (−0.017–0.07), 0.446
	T1 vs. T3	0.071 (−0.021–0.163), 0.197	0.071 (−0.026–0.168), 0.235	0.041 (−0.002–0.085), 0.07
	T2 vs. T3	0.034 (−0.058–0.126), 1	0.011 (−0.086–0.108), 1	0.015 (−0.029–0.059), 1
Between-group comparisons	T1	−0.089 (−0.199–0.022), 0.116	−0.127 (−0.245–0.009), 0.135	0.052 (−0.001–0.104), 0.054
	T2	−0.471 (−0.64– −0.302), 0	−0.487 (−0.664– −0.309), 0	−0.096 (−0.176– −0.016), 0.019
	T3	−0.695 (−0.918– −0.472), 0	−0.711 (−0.946– −0.476), 0	−0.135 (−0.241– −0.029), 0.012

Within the E-CR group, all three biomarkers showed significant reductions from T1 to T2 and from T1 to T3 (all adjusted *p* < 0.001). For example, logNfL decreased by 0.419 units from T1 to T2 and by 0.677 units from T1 to T3, while logGFAP showed comparable declines (0.42 and 0.656, respectively). IL-6 also declined significantly over both intervals. These results suggest sustained and progressive decreases in biomarker levels among patients receiving early rehabilitation. In contrast, the Non-early-CR group showed no significant within-group changes across any time points (all adjusted *p* > 0.05), indicating minimal spontaneous biomarker recovery over 12 months.

Between-group comparisons further supported these findings. While no markers differed at baseline (T1), significant differences emerged by 6 months (T2) and became more pronounced at 12 months (T3), with the E-CR group consistently showing lower levels of logNfL, logGFAP, and logIL-6 (all adjusted *p* < 0.05). These results correspond closely with the patterns shown in Table 7.

These results indicate that early cognitive rehabilitation was associated with faster decreases in serum markers of neuroaxonal injury and inflammation, with between-group divergence becoming evident at 6 months and further widening by 12 months.

### 3.10 Associations between serum biomarkers and cognitive outcomes

To examine whether biological recovery was linked to cognitive improvement, correlation analyses and multivariable linear regression models were performed. As shown in Table 10, all three biomarkers—logNfL, logGFAP, and logIL-6—were negatively correlated with MoCA scores at both 6 and 12 months (all *p* < 0.01). The strongest associations were observed at 12 months, where higher biomarker levels corresponded to lower cognitive performance (e.g., logNfL: r = −0.319, *p* < 0.001).

**Table 10:** Correlation analysis between biomarkers and MoCA scores.

Biomarker	MoCA at 6 months		MoCA at 12 months	
	r	P	r	P
logNfL	−0.241	0.001	−0.319	<0.001
logGFAP	−0.237	0.001	−0.329	<0.001
logIL-6	−0.279	<0.001	−0.341	<0.001

Multivariable regression further supported these relationships (Table 11). Early cognitive rehabilitation (E-CR vs. Non-early-CR Group) remained a significant positive predictor of MoCA scores in all models. However, the magnitude of its association decreased after biomarker levels were added to the model (e.g., 12-month MoCA: *β* reduced from 3.791 to 2.302). This attenuation suggests that part of the cognitive benefit associated with early rehabilitation may be mediated through reductions in neuroaxonal injury and inflammation.

**Table 11:** Multivariable linear regression analysis of total MoCA scores.

Outcome variable	Predictor	Model 1 β (*p* value)	Model 2 β (*p* value)	Model 3 β (*p* value)
MoCA at 6 months	E-CR vs Non-early-CR Group	3.591 (<0.001)	2.821 (<0.001)	2.182 (0.028)
MoCA at 12 months	E-CR vs Non-early-CR Group	3.791 (<0.001)	3.602 (<0.001)	2.302 (0.039)

Overall, these analyses indicate that lower levels of NfL, GFAP, and IL-6 were independently associated with better cognitive outcomes, suggesting a potential biological correlate of the relationship between early rehabilitation and long-term cognitive recovery.

## 4 Discussion

This study, based on a real-world retrospective cohort design, explored the association between E-CR and long-term cognitive recovery in patients with mTBI. The results showed that the E-CR group had greater improvements in MoCA scores at both the 6-month and 12-month follow-up and a higher likelihood of achieving cognitive normalization than the Non-early-CR group. Moreover, the E-CR group also showed substantial improvements in ADL and GOS-E. These findings suggest that implementing structured cognitive intervention in the early stages after mTBI may help promote long-term cognitive and functional recovery, providing clinical support for further prospective evaluation of early intervention strategies. It should be emphasized that the relatively long hospital stays in both groups reflect local admission practices for observation and rehabilitation rather than injury severity, as all enrolled patients met mTBI criteria and those with intracranial hemorrhage or severe polytrauma were excluded.

Further analysis of serum biomarkers showed that NfL, GFAP, and IL-6 levels declined more markedly over time in the E-CR group than in the Non-early-CR group. This phenomenon provides clues for understanding the potential neurobiological correlates of early intervention. The decrease in NfL levels, which is commonly regarded as a marker of axonal injury recovery, and the reduction in GFAP levels, which may indicate reduced astrocyte reactivity, were observed in parallel with better clinical outcomes in the E-CR group and may reflect biological processes associated with recovery (Castano-Leon et al., 2023; Sanchez-Juan et al., 2024). Additionally, the decline in IL-6, an inflammatory cytokine, could indicate the role of early rehabilitation in alleviating the local inflammatory environment. The trends in these biomarkers align with the improvements in cognitive function, suggesting that early cognitive training may contribute to neural structural repair, inflammation regulation, and functional network recovery. While causal relationships cannot be established in this study, this “convergence of biological changes and behavioral outcomes” resonates with prior observations of the neuroplasticity window following mTBI (Zotey et al., 2023). These biomarker findings should therefore be viewed as hypothesis-generating observations that are consistent with, but do not establish, a biological pathway linking early rehabilitation to accelerated neural recovery. Formal mediation analyses in larger prospective studies are needed to test this hypothesis.

Alternative explanations for the biomarker differences should be considered, including unmeasured baseline factors and greater overall therapeutic contact in the E-CR group. Rather than establishing a specific mechanism, our biomarker results are consistent with the hypothesis that early cognitive engagement is associated with accelerated resolution of neuroaxonal injury and inflammation. This interpretation aligns with recent randomized evidence. Structured cognitive training improved cognition over active control in chronic mTBI (Mahncke et al., 2021), while targeted treatment and structured behavioral management yielded comparable benefits in the acute-to-subacute phase, suggesting the therapeutic context itself plays an important role (Kontos et al., 2026). Similarly, no specific modality demonstrated superiority in our study, supporting the view that early structured cognitive challenge, rather than a particular module, may be associated with the observed benefits. The specific neurobiological mechanisms require further investigation.

The low proportion of patients achieving MoCA ≥26 in both groups warrants careful interpretation. The mean education level in our sample was approximately 8–9 years, substantially lower than that of populations in whom this cutoff was originally validated. Among Chinese healthy adults with similarly limited education, mean MoCA scores are approximately 23, with age and education explaining nearly half of the variance (Wei et al., 2024). Furthermore, 74–79% of cognitively healthy Chinese adults aged 65–79 years score below 26, a disparity completely explained by education and vascular risk factors (Ke et al., 2025). In addition, the wide age range in our sample further limits the applicability of a single cutoff, as advanced age independently lowers MoCA performance. Notably, age was not a significant predictor of ΔMoCA in multivariable analysis, confirming that E-CR-related improvement was age-independent. Thus, the low rate of MoCA ≥26 observed here likely reflects the demographic norm rather than disproportionate post-mTBI impairment. Future studies should employ education-adjusted thresholds.

Clinically, this study contributes additional evidence to the field of cognitive rehabilitation in mTBI. Most previous studies have focused on moderate to severe TBI populations, with limited attention to mTBI. This study demonstrated that the E-CR group exhibited more significant cognitive recovery and better ADL and GOS-E outcomes, indicating that even mild TBI patients may benefit from structured and continuous cognitive rehabilitation. Furthermore, the dose–response relationship observed between the number of rehabilitation sessions and cognitive improvement suggests that the intensity of rehabilitation may be crucial for efficacy, which is consistent with previous findings that higher rehabilitation intensity correlates with better functional recovery (Fure et al., 2021). However, no significant differences were observed between the different cognitive training modules, suggesting that the key factor in the early stages of mTBI recovery may not lie in the specific training module but rather in the timing and structured cognitive challenge provided.

Although this study has several strengths, several limitations should be acknowledged. First, the retrospective design may have introduced selection bias and residual confounding, as factors such as socioeconomic status, access to rehabilitation services, family support, patient motivation, and rehabilitation adherence were not systematically recorded. Second, the MoCA and Barthel Index may not fully capture domain-specific cognitive deficits or subtle functional impairments in patients with mTBI. Third, the individualized rehabilitation program reflected real-world practice but limited the ability to determine which specific components were most strongly associated with outcomes. Fourth, imaging assessment was based on a single non-contrast head CT within 24 h after injury, and MRI was not routinely performed. Therefore, subtle microstructural injuries, dynamic radiological changes, and lesion location could not be evaluated. Finally, the Non-early-CR group included patients without cognitive rehabilitation and those who initiated rehabilitation after 30 days. Because detailed delayed-rehabilitation records were incomplete, the exact proportion and intensity of delayed rehabilitation could not be reliably determined, limiting the distinction between intervention timing and overall rehabilitation exposure.

## 5 Conclusion

In conclusion, this real-world cohort study suggests that early cognitive rehabilitation is associated with significant long-term improvements in cognitive performance, functional independence, and global recovery among patients with mild traumatic brain injury. The concurrent decline in serum biomarkers of neuroaxonal injury and inflammation suggests that early structured cognitive engagement may contribute to neural stabilization and recovery, although definitive causal mechanisms remain to be determined. These findings support the potential value of initiating cognitive rehabilitation within the early post-injury period and highlight the need for greater clinical attention to structured follow-up and timely intervention in mTBI care. Future multicenter, prospective studies incorporating multimodal neurobiological measures are warranted to validate these results and clarify the relationship between early cognitive rehabilitation, biomarker changes, and neural or functional recovery.

## Data Availability

The original contributions presented in the study are included in the article/supplementary material, further inquiries can be directed to the corresponding author.
